# Beyond dirty teeth: Integrating dental calculus studies with osteoarchaeological parameters

**DOI:** 10.1016/j.quaint.2022.03.003

**Published:** 2023-04-20

**Authors:** Anita Radini, Efthymia Nikita

**Affiliations:** a BioArCh, Department of Archaeology, The University of York, Wentworth Way, York, UK; b York JEOL Nanocentre, The University of York, Science Park, York, UK; c Science and Technology in Archaeology and Culture Research Center, The Cyprus Institute, 2121, Aglantzia, Nicosia, Cyprus

**Keywords:** Dental calculus, Osteoarchaeology, Life history, Environment

## Abstract

The study of ancient human dental calculus (mineralized dental plaque, also known as tartar) is becoming increasingly important in osteoarchaeology, human palaeoecology and environmental archaeology. Microremains of different origin (e.g. starch granules, pollen, phytoliths, feather barbules) as well as biomolecules and chemical compounds retrieved from its mineral matrix may represent an important link between past humans and their physical, biological and social environment, but they are rarely fully linked to the evidence from skeletal remains. This paper critically reviews the lines of evidence retrieved from dental calculus in relation to osteoarchaeological parameters, employing macroscopic, microscopic and biomolecular approaches, assessing synergy potential and limitations. The scope of this paper is also to contribute to the building of a much needed theoretical framework in this emerging subfield.

## 1 Introduction: enhancing resolution in osteoarchaeological research through dental calculus analysis

Osteoarchaeology^1^ focusses on the study of human skeletal remains from archaeological contexts and offers invaluable information regarding diet, mobility, pathology, activity, demography and other aspects of life in the past (Larsen, 2015). The capacity of the human skeleton to act as a valuable reservoir of information is based on the dynamic nature of the skeletal tissues that remodel throughout an individual's lifetime in response to the living conditions (e.g., differential access to dietary resources, exposure to pathogens, activity levels, and others) (Buikstra and Beck, 2017; Martin et al., 2013; Nikita, 2017). Research questions pertaining to the biocultural adaptation of past humans to the natural and socio-cultural environment hold a central position in modern osteoarchaeological inquiry, acknowledging that the biological and socio-cultural identities of humans are inextricably linked (Temple and Stojanowski, 2018; Zuckerman and Armelagos, 2011). Despite its potential to elucidate highly diverse aspects of past life through the most direct evidence of the individuals who experienced it, their bones, osteoarchaeology suffers from various methodological and interpretational limitations. A central limitation is that most osteoarchaeological data offer only indirect information regarding life in the past. As a typical example, through the study of human bones, we can only indirectly deduce what food people consumed. Dental (micro)wear offers information regarding how tough or processed food was (Schmidt et al., 2019), isotope analysis reveals the relative importance of plants with different photosynthetic pathways and marine versus terrestrial resources (Bogaard and Outram, 2013), while dental diseases such as caries provide insights to the consumption of carbohydrates (Humphrey et al., 2014). Nonetheless, none of these methods can reveal some of the specific foods that people were actually consuming; instead, they provide important but indirect evidence regarding past dietary practices. Similarly, different approaches have been used to reconstruct past occupations based on skeletal remains, with most prominent in the literature the study of long bone cross-sectional geometric properties and entheseal changes. The former express the skeleton's biomechanical adaptation to torsional, compression or bending forces (Ruff, 2018), while the latter represent morphological changes at the sites of muscle/tendon/ligament attachment on the skeleton (Villotte and Knüsel, 2013). Even though these methods offer information regarding the direction and nature of mechanical stresses applied on the skeleton and the muscles that were systematically used, they cannot reveal the exact occupations in which individuals engaged; hence occupational reconstruction has been deemed ‘Bioarchaeology's Holy Grail’ (Jurmain et al., 2012). This lack of specificity limits our potential to disentangle the dynamic interaction between past humans and their physical, biological and social environment.

**Fig. 1:**
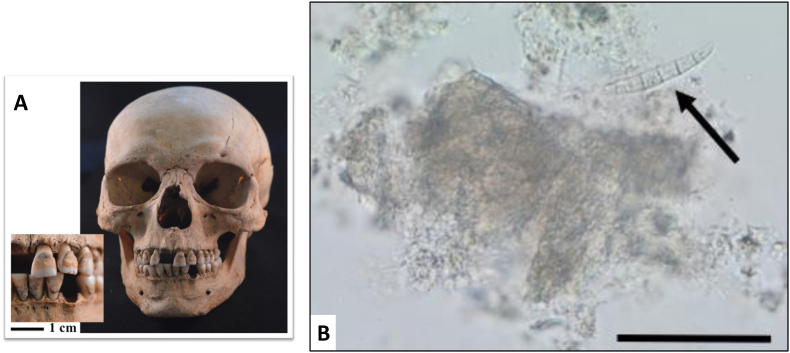
**A:** Dental calculus deposit on the teeth of individual sk218, St Michael's Parish, Medieval Leicester (1250–1400 AD), picture by Radini (2016)**B:** Pseudo *in situ* fungal debris (arrow pointing at cf. *Fusarium* sp.) in the process of being ‘freed’ from an ancient calculus fleck (the pale yellowish brown mass surrounding the fungal debris). Scale bar: 50 μm. Picture by Radini (2016), from a Medieval individual from Leicester UK. (For interpretation of the references to colour in this figure legend, the reader is referred to the Web version of this article.)

Dental calculus is mineralized dental plaque adhering on the teeth, and holds considerable potential to refine osteoarchaeological information (Fig. 1 A). It is formed by the activity of bacteria in the mouth and, if not removed as soft plaque, it can become mineralized in as little as two weeks (Lieverse, 1999, 220). While forming, calculus can entrap particles of different nature, including starch granules, phytoliths (plant opal), fungal debris (Fig. 1 B), microscopic insect parts but also human cells and mineralized bacterial structures, and chemical compounds (e.g., Blondiaux and Charlier, 2008; Charlier et al., 2010; Dobney and Brothwell, 1987; Fox et al., 1996; Hardy et al., 2012). Moreover, human dental calculus is also a very important source of biomolecular information, including proteins, DNA and metabolites associated with the human oral microbiome and salivary/oral proteome (Adler et al., 2013; De La Fuente et al., 2013; Hendy et al., 2018; Preus et al., 2011; Warinner et al., 2014; Wright et al., 2021). Initially, dental calculus research largely focused on dietary evidence (see Blondiaux and Charlier, 2008; Radini et al., 2017). Since then, it has been shown that microdebris and chemical compounds that are not of dietary origin may be preserved in the calculus matrix. Blatt et al. (2011) recovered evidence of cotton fibers (*Gossypium* spp.) *in situ* in the dental calculus from skeletal remains from the site of Danbury, dated to 900–1100 CE, representing the first evidence of this fiber in Ohio. Blondiaux and Charlier (2008), in their study to assess the forensic potential of dental calculus, found several types of mineral debris, and elemental analysis of some of these crystals allowed them to detect possible work-related intoxication in one individual from the Etruscan population of Monterenzio Vecchia. Radini et al. (2019) retrieved evidence of pigments from the dental calculus of a Medieval female. Moreover, the identification of specific chemical compounds has allowed Hardy et al. (2012) to detect exposure to smoke and bitumen in individuals of the Neanderthal population unearthed at El Sidrón, Spain. Therefore, a variety of microdebris and chemical compounds may be recovered from the dental calculus matrix that can inform us about natural and built environments. The survival of evidence generated by human activities other than eating, also highlights that several pathways are possible to the inclusion of microdebris and molecules in human dental calculus apart from diet, which may complicate the interpretation of relevant evidence (Radini et al., 2017).

From the above brief outline, it becomes clear that particles and molecules retrieved from dental calculus offer the possibility to elucidate aspects of past life with a resolution that expands that of osteoarchaeology as they allow a direct link with food and environments experienced by individuals during life. This paper critically reviews the lines of evidence retrieved from dental calculus, with a strong focus on microremains, in relation to osteoarchaeological parameters, contributing to the building of a theoretical framework in this emerging subfield, which intercrosses osteoarchaeology, human palaeoecology and environmental archaeology.

## 2 Aims and structure of the paper

### 2.1 Aims

In our recent work (Radini et al., 2017) we demonstrated how microscopic remains embedded in dental calculus can provide broader information than diet alone, and highlighted the importance of careful extraction and identification of microdebris particles. The current paper emphasizes the need to contextualize the remains by integrating them with other osteoarchaeological parameters from the skeletons from which they originate. As the potential of this still rather new data becomes clearer, the need for a theoretical framework and a better understanding of methods to be applied, has also increased. Recent methodological work on dental calculus has focused on refining protocols for microfossil extraction, ensuring no contamination occurred post mortem (e.g. from burial soils) or post excavation (e.g. in the laboratory) (e.g. Cristiani et al., 2016; Le Moyne and Crowther, 2021), as well as implementing biomolecular analysis (Farrer et al., 2021; Palmer et al., 2021; Wright et al., 2021). In addition, a few literature reviews have discussed the potential and limitations of microdebris and biomolecules retrieved from calculus in broader critical terms (e.g. Hendy et al., 2018; Radini et al., 2017; Wright et al., 2021).

Here, we focus on the need to strengthen the links between the information obtained from skeletal remains and from dental calculus with a double goal of expanding and testing methodological work and maximizing the retrieved information. While we focus on microremains, we also make reference to key biomolecular and chemical work. This is particularly important when we consider the need to be effective in the way we target and use the limited amount of dental calculus available from the dentitions of past individuals, reassessing the need of more sustainable dental calculus research in archaeology (Mackie et al., 2017; Radini, forthcoming). This paper builds upon the taphonomic processes illustrated in our previous work (Radini et al., 2017), it aligns with Bucchi's et al. (2019) call for a discussion on the role of ancient human dental calculus research in archaeology, and highlights ways to interlink dietary, pathological and occupational information obtained from osteoarchaeology and dental calculus research so as to gain a more holistic understanding of past life from a single individual to the population level. Note that the potential contamination of samples is a key issue in dental calculus research, as very well detailed in Warinner et al. (2014). For the scope of this work, we trust that the calculus data published in the quoted papers have the necessary archaeological integrity not to be considered the result of contamination. We however stress that a better understanding of the mechanism of inclusion of potential contaminants, from the interaction of the burial soil fungi with dental calculus to the movement of molecules from the soil into the porosities of the calculus itself, is a key issue in this field, which will need to be fully addressed in the future.

In promoting a more solid methodological bridge between the field of Osteoarchaeology and that of Dental Calculus research, this paper extends to dental calculus research the concept proposed by Shillito (2017) of multi-scale integrated approaches ‘to fill the gap between macro- and micro-archaeological methodologies’ in other to create an improved multiproxy archaeological analysis in the study of the past. We hope that relevant considerations may be adopted by biomolecular specialists, though this goes beyond the aims of this paper.

### 2.2 Structure

The paper is divided into broad thematic categories covering the main aspects of past life elucidated by means of osteoarchaeology where dental calculus research can make an important contribution: food access and diversity, natural environment, pathology, occupation, and life course approaches. For each of the broad topics discussed, we outline the information provided by osteoarchaeology, employing macroscopic, microscopic and biomolecular approaches, followed by a critical analysis of existing data from dental calculus research, assessing synergy potential and limitations.

## 3 Combining osteoarchaeological parameters with dental calculus

### 3.1 Food: access and diversity

Food can be approached from many standpoints; nutritional, economic, social, religious and even ecological (Goody, 1982; Livarda, 2008, 2011; Twiss, 2012; Van der Veen, 2003). Access to food (quantity, quality and variety of foodstuffs) is a key factor in human wellbeing and a core research area in archaeology. The Archaeology of Food is a thriving field and has generated a variety of datasets to study past foodways (Cutright, 2021; Twiss, 2019). Here, we contrast the evidence of diet and food access available from osteoarchaeological research with that of the dietary evidence retrieved from dental calculus, assessing how the two fields can be better integrated.

#### 3.1.1 Osteoarchaeological evidence on past diet

Dietary reconstruction through osteoarchaeological remains is indirect and employs macroscopic, microscopic and biochemical methods. Macroscopically, ancient diet can be approached through the relative frequency of dental pathologies (Flensborg et al., 2019; Mayes, 2016). Traditionally, dental caries has been associated with increased consumption of carbohydrates (Koca et al., 2006; Temple and Larsen, 2007), whereas dental calculus deposits have been linked to protein consumption (Lillie, 1996). Therefore, the frequency of these dental conditions can offer insights to the relative importance of protein and carbohydrates in the diet. Nonetheless, it is acknowledged that dental caries and calculus are multifactorial in etiology; many parameters beyond dietary practices (e.g. salivary flow rate, oral hygiene, hormones) affect their expression and different foodstuffs may interact and influence the appearance of the above conditions in complex ways (for example, milk products containing the protein casein have a cariostatic effect) (Lieverse, 1999; Lukacs, 2008; Lukacs and Largaespada, 2006; Mundorff-Shrestha et al., 1994; Shearer et al., 2011; Shuler, 2001).

Certain palaeopathological conditions identified on past human skeletal remains may also offer indirect dietary information by pointing towards the insufficient intake of specific nutrients. A typical example is vitamin C deficiency, which results in scurvy. As humans cannot synthesize their own vitamin C, they rely on dietary intake with the main sources of this vitamin being fresh fruit and vegetables, followed by meat, fish, and dairy products. Deficient vitamin C results in reduced bone formation, which manifests as osteopenia and hemorrhage-induced subperiosteal new bone formation and/or cortex porosity in subadults and in adults, though in the latter bone changes are much subtler (Brickley and Mays, 2019). The limitations of using palaeopathological data to reconstruct past diet include the lack of specificity of many pathological lesions (i.e. a secure diagnosis of scurvy may often be problematic unless the skeleton is well preserved and manifests pathognomonic lesions) and the fact that even if a secure diagnosis is reached, it only supports that the individuals under study likely did not consume sufficient amounts of fresh fruit and vegetables; it does not specify what kind of food they did consume. Beyond scurvy, iron-deficiency anemia has also been identified on skeletal remains and is linked to a diet deficient in iron (e.g. McIlvaine, 2015), while non-specific physiological stress markers, such as enamel hypoplasia, may also be associated to dietary deficiencies (Folayan et al., 2020), though in these cases as well, several other causative factors should be seriously considered (e.g. Anthonappa and King, 2015; Walker et al., 2009).

Dental wear has also been used as a means of exploring dietary patterns in archaeological assemblages (e.g. Fiorenza et al., 2018). Relevant approaches are based on the premise that the mechanical and physical properties of the consumed food will affect the chewing process and this will be reflected in the wear patterns seen on the dental surfaces (Watson and Schmidt, 2020). Methodologies to macroscopically examine dental wear include the use of ordinal systems which express the degree of lost enamel and/or exposed dentine (Scott, 1979; Smith, 1984), quantitative approaches that measure the surface area of exposed dentine (Clement and Hillson, 2012), and 3D dental wear pattern analysis (Occlusal Fingerprint Analysis) (Silvester, 2021). The limitation of such methods is that dental wear is an indirect approach to reconstructing past diets; it may offer insights to subsistence practices, dietary composition and exogenous abrasives, and food processing, while when compared among individuals at an intra- or inter-cemetery level, it may offer interesting information on sex-, age- or social-related dietary biases. However, it cannot specify what food was consumed, while it is also affected by non-masticatory uses of the teeth, oral hygiene, inherited dental tissue quality and other factors (Watson and Schmidt, 2020).

The same limitation applies to the microscopic analysis of dental wear, known as dental microwear analysis (Teaford, 1994). Such analysis traditionally examined the relative frequency of pits and scratches and subsequently drew conclusions regarding the degree of food processing, the presence of abrasive inclusions in consumed items, as well as the hardness of the food itself (Schmidt, 2001; Ungar and Spencer, 1999). More recent approaches explore the topography of the tooth surface (surface texture analysis) and allow a more complex quantification of the masticatory surfaces (Schmidt et al., 2019). A key limitation of dental microwear analysis in human osteoarchaeological assemblages is that ancient diet and food producing and processing strategies are particularly varied and it is extremely difficult to decipher this variation through microwear patterns. An additional issue is the use of teeth as tools in a wide range of activities, which further complicates the interpretation of microwear patterns. This is why dental microwear has been particularly employed in zooarchaeology (Jiménez-Manchón et al., 2019), primatology (Ragni et al., 2017) and paleoanthropology (Williams et al., 2018), where the environmental niche, access to resources and extra-masticatory activities are somewhat more easily controlled.

With regard to biochemical methods, stable carbon and nitrogen isotopes have been widely used in palaeodietary analysis (Bogaard and Outram, 2013). In brief, plants differ in their stable carbon isotope (δ^13^C) values depending on the photosynthetic pathway they follow. Most terrestrial plants use the C3 pathway, tropical grasses mainly use the C4 pathway, while cacti and succulents follow the Crassulacean Acid Metabolism (CAM) pathway. As animals consume plants, the stable carbon isotope values in their tissues vary according to their diet. Besides providing information on plant consumption, carbon isotope values also show the extent to which diet was primarily based on marine or terrestrial resources since such resources exhibit very different values to each other (DeNiro and Epstein, 1978; Killgrove and Tykot, 2013; Müldner, 2013; Van der Merwe, 1982). Stable nitrogen isotopes (δ^15^N) also offer important information regarding protein consumption. The value of these isotopes differs depending on the trophic level of the consumed animals and are higher in marine organisms compared to terrestrial ones (Ambrose, 1991; DeNiro and Epstein, 1981; Hedges and Reynard, 2007; Schoeninger and DeNiro, 1984). Nitrogen isotopes are also useful in exploring past breastfeeding and weaning practices; breastfed children have δ^15^N values slightly higher than their mothers and these values decrease once the weaning process begins until they become equated to the value of solid diet (Fogel et al., 1989; Schoeninger and DeNiro, 1984; Tsutaya and Yoneda, 2015). A key limitation of dietary isotopic analysis is that it provides indirect information regarding plant and animal resources. For instance, it may suggest a clear dominance of C4 plants in the diet but it does not specify which C4 plants were consumed. Furthermore, when a population's diet is diverse, it is difficult to distinguish among C3, C4, marine and freshwater consumption due to overlapping bulk carbon isotopic values (Webb et al., 2015). Similarly, diet to tissue fractionation, aridity levels, manuring practices, nutritional stress and other factors can complicate trophic level reconstructions based on δ^15^N values (Fraser et al., 2011; Fuller and Petzke, 2017). Given the above limitations associated with bulk isotope data, the advancement of single compound carbon (δ^13^C) and nitrogen (δ^15^N) isotope analyses has enhanced the resolution of dietary reconstructions based on these isotopes given the different metabolic pathways of individual amino acids in bone collagen (Itahashi et al., 2020; Ma et al., 2021; Naito et al., 2016; Webb et al., 2018).

#### 3.1.2 Dental calculus dietary evidence

Dental calculus research has placed a huge focus on the retrieval of plant debris from calculus deposits to gain information on diet, especially deep in the human past (e.g. Cristiani et al., 2018; Henry and Piperno, 2008; Henry et al., 2012, 2014; Horrocks et al., 2014; Li et al., 2010; Madella et al., 2014; Mickleburgh and Pagán-Jiménez, 2012; Nava et al., 2021; Piperno and Dillehay, 2008; Tao et al., 2015; Wang et al., 2016; Wesolowski et al., 2010). To date, from the point of view of light microscopy, secure evidence of deliberate consumption of food of animal origin remains very limited and mainly consists of fish scales from Mesolithic Balkan calculus samples (Cristiani et al., 2018). The application of biomolecular techniques to dental calculus holds some potential for the identification of DNA and proteins of animal origin, as attested by the retrieval of evidence of milk consumption (e.g. Charlton et al., 2019; Warinner et al., 2014), however this may be limited (Hendy et al., 2018). In addition, some fungal spores have been found in dental calculus and may be interpreted as food (e.g. Hardy et al., 2018) but due to limitations in identifying remains from the Kingdom of Fungi (MacKenzie, 2021; Radini et al., 2017), it is not yet possible to link these remains to deliberate mushroom consumption.

Despite the great potential of dental calculus deposits to preserve food items originating from the Plant Kingdom, a number of issues have been raised regarding the interpretation of such remains. The first important aspect to take into account is how representative the evidence of plant remains retrieved from dental calculus analysis is with respect to the role the ingested plant material played in the diet of the individuals (in terms of quality, quantity and frequency of ingestions). Ethnographic research on modern populations has shown staple starchy food, such as maize, eaten once a day was only found in 30% of the calculus samples of the individuals who took part in the study (Leonard et al., 2015). More recently, research by Power et al. (2021) has shown that phytoliths too are underrepresented in dental calculus material. These studies show that starch and phytoliths, the two key typologies of plant remains found in dental calculus, do not reflect the variety of plant food ingested. In terms of quantity, it is also impossible to correlate small amounts of starch granules to the actual quantity of ingested food (Hardy et al., 2018). The second issue is of methodological nature and concerns the extraction of the microremains from calculus. It is becoming increasingly clear that some chemical compounds commonly used to ‘disaggregate’ the calculus matrix in order to extract microremains may have damaging effects on the microremains themselves and can create biases in their retrieval (Le Moyne and Crowther, 2021; Tromp et al., 2017). Further experimental work and research is needed to clarify what portion of the diet is visible in dental calculus and how methodologies of extraction of remains can be improved. All above issues have been discussed in recent reviews and methodological work in the field and we refer to them for further in depth reading (see Hardy et al., 2018; Power et al., 2021; Radini et al., 2017). Nevertheless, despite the above limitations, there is widespread agreement in the dental calculus research community that this deposit offers great potential to provide new insights about ingested items in ancient individuals, especially plant remains (Hardy et al., 2018).

#### 3.1.3 Integrating dental calculus microscopy with osteoarchaeological data on past diet

We have identified three interconnected areas where dental calculus research and osteoarchaeology can most productively complement each other; here we will discuss *food diversity* and *food access,* while the subject of *food hygiene* will be explored in the section of oral health for its potential connection to dental wear and oral pathologies.

*Food diversity.* Through the identification of starch granules, phytoliths and other plant parts, dental calculus can reveal a variety of plants consumed in the past, thus, helping osteoarchaeological dietary reconstruction move past the limits of identifying generically a high reliance on starchy food (through high caries rates) or C3 vs. C4 contributions (through isotope analysis). Hardy et al. (2018) suggested that a critical understanding of the evidence from diet retrieved in calculus is of paramount importance in the reconstruction of past diet. A critical overview of dental calculus data from plants, shows that dental calculus not only records staple food plants, such as members of the Triticeae Tribe (the tribe of barley and wheat) and their ancestors (such as *Aegilops* spp.), but it also gives visibility to food crops such as oats (Tribe Avenae) and legumes (members of the family Fabaceae) that traditionally have lower archaeological visibility, potentially due to the fact that they came less often in contact with fire when compared to cereals (Hall, 2001; Moffett, 2011). Another important category of remains that was until recently problematic in the archaeological record is that of millets (Madella et al., 2014). The category for plants currently grouped under ‘millets’ encompasses a wide range of wild (e.g. *Brachiaria deflexa*) and domesticated (e.g. *Panicum miliaceum*) species that are an important food crop in many parts of the world, as well as weeds of arable fields (Madella et al., 2016). In recent years, microscopic analysis of dental calculus and stone tools in different regions and periods of time has provided a solid record for the consumption of wild and domesticated species of millets even where there is limited or no archaeobotanical evidence for any members of this important Tribe of plants (e.g. Lucarini et al., 2016; Lucarini and Radini, 2020). Although the formation process of dental calculus is still not well known, it is becoming clear that this deposit has the ability to entomb microremains from plants that have lower archaeological visibility when compared to the staple cereal crops (such as wheat and barley, C3 plants), as is the case of millets (C4) but also legumes (e.g. Cristiani et al., 2016; Hardy et al., 2016; Li et al., 2010; Lippi et al., 2017; Madella et al., 2014; Tao et al., 2015; Zanina et al., 2021). This is probably due to the very different preservation environment of dental calculus when compared with archaeobotanical evidence retrieved in soil. Indeed, work conducted on artificially charred grains has demonstrated that grains of domesticated species of millets do not have the same survival rate as other larger and tougher cereal grains, supporting that the lower visibility of millets in the archaeobotanical record could be the result of preservation issues (e.g. Colledge and Conolly, 2014). Therefore, the combination of dental calculus plant microdebris with isotopic analysis can promote understanding of how different groups of plants contributed to diet (see case study in Tao et al., 2015).

Another category of remains which greatly enhances our understanding of the diversity of available foodstuff, is that of imported species. A growing body of evidence is showing that dental calculus microscopy analysis, especially in combination with biomolecular techniques, can reveal the consumption of exotic plant species, which are also very rarely retrieved in traditional archaeobotanical analysis from soil specimens. Exotic plant species from the New World were identified in individuals of Victorian Period Ireland and supported increased consumption of relief food,maize (*Zea mays*) and potato (*Solanum tuberosum*), during the Great Famine (1845–1852) (Geber et al., 2019). More recently Scott et al. (2021) retrieved evidence of imported species of plants during the Bronze Age in the Eastern Mediterranean, including sesame, although via proteomic analysis. Finally, MacKenzie et al. (in press), retrieved evidence of New World crops, in specific maize or Indian corn (*Zea mays*) and tapioca (*Manihot esculenta*), in individuals from Post-Medieval Manchester. These are strong examples that demonstrate how dental calculus can provide unique insights into plants that are new ‘culinary’ arrivals and would be challenging to retrieve from the archaeobotanical records of soil alone. Data from plants in dental calculus are clearly complementary to those of plants recovered from the soil and complementary to isotopic analysis on human bones, even if they do not reflect the full range of plant food ingested and its relative quantity. As such, dental calculus data would be particularly interesting to also combine with evidence for ancestry and migration, including the analysis of biodistances (employing dental or cranial nonmetric traits, or craniometric data) and ^87^Sr/^86^Sr and δ^18^O isotope measurements on the dental enamel. Such osteoarchaeological analyses offer insights to past mobility patterns (gene flow and kinship in the case of biodistance analysis; discrimination between local or non-local individuals in the case of ^87^Sr/^86^Sr and δ^18^O isotopes analysis) (Bentley, 2006; Pederzani and Britton, 2019; Pietrusewsky, 2014). The combined study of palaeomobility and the consumption of imported species can elucidate key aspects of differentiated ‘ethnic’ dietary preferences, and the degree to which these are preserved and/or spread to other groups after relocation.

*Food access.* Strongly interlinked with the theme of food diversity and the consumption of imported species, is the theme of food access, that is, the variety, type and quality of food consumed across past populations. At the moment, there is little synthetic work on plant foodstuffs in their archaeological and social context, and it is difficult to assess which foods could be accessed by different individuals based on the traditional archaeobotanical record (Moffett (2006, 55). This very serious limitation could be at least partly overcome by combining the study of imported/luxury items in dental calculus with various osteoarchaeological parameters. Associations with sex and age-at-death data could indicate gender (with the serious potential pitfall of equating sex with gender) or age-related status differences within past populations. Moreover, associations with skeletal evidence of physiological stress (e.g. linear enamel hypoplasia - Ham et al., 2021), skeletal markers of increased mechanical strain, suggestive of individuals involved in physically demanding occupations (e.g. osteoarthritis, Schmorl's nodes, entheseal changes - Eng, 2016; Karakostis et al., 2019; Üstündağ, 2009), or poor health (e.g. anemias, metabolic disorders, infectious diseases - Brickley and Mays, 2019) could elucidate important aspects of past social stratification and the biological manifestation of social inequalities. Finally, as mentioned in the previous section, combining the analysis of imported and/or luxury food items retrieved in dental calculus with biodistance or isotopic analysis of palaeomobility, could reveal whether there was differential food access between locals and non-locals but also to what extent new dietary habits spread in new locations.

We acknowledge that limitations in the secure identification of imported plant species or other socially and economically important foods may reduce the applicability of the proposed approaches. We encourage experimental work on reference collections to assess what key plant foods survive cooking and other types of processing, and are sufficiently preserved so as to be identified in the small fragments that can be captured in dental calculus. This has been routinely conducted on starchy foods, such as legumes and cereals (Barton and Torrence, 2015), but has not been applied systematically to other lines of evidence and to parts of plants that are not starch or phytoliths. In addition, the above listed osteoarchaeological markers of potential social status (skeletal occupational markers, markers of physiological stress etc.) suffer from inherent limitations mostly linked to the fact that their expression is multifactorial (Domett et al., 2017; Edinborough and Rando, 2020; Michopoulou et al., 2015); hence, they should be used critically and in combination when assessing status. Ideally, such skeletal markers should also be used in conjunction with material cultural evidence of social status as a complementary line of evidence (though with its own inherent biases) (Robb et al., 2001). Similarly, palaeomobility studies also have inherent limitations, such as the inability to securely identify the point of origin of individuals characterized as non-locals by isotope analysis (Bentley, 2006) or the fact that biodistance analysis only offers broad insights into past mobility (Nikita, 2020). The amplification of studies developing baselines of bioavailable Sr at different regional scales (e.g. Bataille et al., 2020; Hoogewerff et al., 2019; Ladegaard-Pedersen et al., 2020) and the adoption of even more advanced computational approaches in biodistance analysis, often drawn from genetic studies (Relethford, 2016), aim at addressing these issues but, for the time being, these limitations should be seriously considered at the stage of research design and during results interpretation.

### 3.2 Natural environment

#### 3.2.1 Osteoarchaeological evidence on past environment

As Derevenski (2001) highlights, osteoarchaeology and environmental archaeology may be often seen as distinct research fields; however, characteristics that superficially appear as distinctive between them are actually unifying. As explained above, osteoarchaeology examines past human remains in their physical and sociocultural environment (Larsen, 2015). This environment controls exposure to diseases, availability of dietary resources, and other parameters that affect human biology. At the same time, the environment is constantly being manipulated and transformed by humans. This human-environment interaction is complex and multifactorial (Derevenski, 2001). As such, human remains from archaeological sites serve as an important source of archaeological evidence to address questions about human-environment interactions, subsistence practices, and their consequences for human populations since natural and social environments leave their mark on the human skeleton (Robbins Schug, 2011).

Despite the very strong interconnection between osteoarchaeology and environmental archaeology, the evidence one may extract from the human skeleton towards reconstructing past natural environments is minimal. Instead, there has been strong interest towards the biocultural impact of climate change in different temporal and geographical settings by means of changes in demographic patterns, growth patterns, physiological stress, diet, health and violence. As a representative example, Robbins Schug (2011) explored the impact of climate change on the subsistence and health of human populations during the latter half of the second millennium BC in peninsular India employing demographic, growth, dietary and pathological data, and highlighted the complexity between environmental conditions, dietary adaptations, and biocultural outcomes. On a more diet- and mobility-focused level, Gregoricka (2016) examined the impact of increased aridification at the end of the third millennium BC on Bronze Age populations in the Oman Peninsula. Encompassing δ^18^O data from human dental enamel, the author confirmed this aridification event, while strontium and carbon isotope ratios suggested continuity in community composition and diet throughout the late third to early second millennium BC, which highlighted the ability of local communities to adapt to changing environmental and cultural conditions. In a study combining dietary data with violence patterns, Tung et al. (2016) examined the impact of Imperial decline and severe drought in the ancient Peruvian Andes with a focus on the juvenile population. As a final example, Harrod and Martin (2014) explored the connection between climate change and violence, and proposed a so-called ‘Biocultural Model for Multicausal Pathways to Increased Violence’, which takes into account environmental degradation, inequality, migration, fear of unpredictable shortages, ethnic identity, social fragmentation and climate change in generating violent conflict. All above studies correctly stress the complex interrelationship between the natural environment, and the biological and social responses of humans to it.

#### 3.2.2 Dental calculus evidence concerning the natural environment

There is a wide range of microremains retrieved from calculus, though often in small numbers, that can provide direct evidence of species of plants and animals surrounding past individuals. Pollen found in dental calculus can be divided into two key categories: trees/shrubs and herbaceous plants. Pollen from trees is more common than that of herbaceous plants and normally belongs to wind dispersed species, such as birch (*Betula* spp.) and pine (*Pinus* spp.) (e.g. Cristiani et al., 2016; Hardy et al., 2016), which would be very common in the environment. Herbaceous plants have been found in a variety of contexts and are mainly represented by edible plants, such as mauve (Malvaceae) from Mesolithic samples from the Balkans (Cristiani et al., 2018) or grasses and weeds of crops (e.g. Radini et al., 2016). Therefore, pollen retrieved from dental calculus is potentially very useful in order to understand the natural environment, in similar ways to those discussed by Shillito et al. (2020) in their work on coprolites. In addition, it is particularly relevant to discuss the evidence of phytoliths in dental calculus. Phytoliths are routinely retrieved in dental calculus studies where they have been often linked to food consumption (Radini et al., 2017). However, recent work by Tromp and Dudgeon (2015) has shown that their interpretation is more complicated than thought. In their previous work at Rapa Nui, Dudgeon and Tromp (2014) had retrieved evidence of large amounts of phytoliths from species of palm that were considered extinct at the time during which the studied individuals lived. The evidence puzzled the authors, who conducted experimental work and proved that the phytoliths from the extinct palms had become entrapped in dental calculus as a consequence of the consumption of tubers. Growing in soil, the tubers had incorporated phytoliths into their peel and external layers belonging to extirpated plants from past environments. Another interesting category of microdebris is diatoms, which may provide useful information on water quality and environment (Dudgeon and Tromp, 2014). Finally, feather barbules, fungal spores and microcharcoal have also been found in calculus, and if identified to species or even Family level, they can provide important information on the natural environment; however, if not correctly identified, it is hard to determine their origin and their link to the environment (e.g. Afonso-Vargas et al., 2015; Cristiani et al., 2018; Juhola et al., 2019; Radini et al., 2016). Therefore, the careful recording of all lines of evidence in calculus and their critical examination at individual and at population level can potentially provide some indication of the natural (and built) environment experienced by ancient people during life.

#### 3.2.3 Integrating dental calculus microscopy with osteoarchaeological data on past natural environment

At the time of writing this review, no study to our knowledge had been published exploring environmental parameters deduced from dental calculus and their biocultural impact as assessed through osteoarchaeology, in part due to the lack of data sets sufficiently large to allow meaningful inferences. Given the highly complex interaction between natural and cultural environments and their biocultural impact, such integrated studies could enhance our understanding of the dynamic and context-specific interplay between humans and their surroundings. A comprehensive study of this interplay would require secure identification of microremains in calculus and their pathway to inclusion, as well as many more lines of evidence to be pulled together, especially from palaeoclimatology, zooarchaeology and archaeobotany.

### 3.3 Pathology

The retrieval in dental calculus of potential medicinal plants (e.g. Fiorin et al., 2019) as well as respiratory irritants, such as microcharcoal and fungal spores (e.g. Hardy et al., 2016; Radini et al., 2016), opens the exciting possibility to better understand ‘ill health’ and the medicinal use of plants in relation to specific pathologies in past populations. Despite the potential of this approach, some hidden limitations need careful consideration. This section of the paper examines the potential and limitations of dental calculus microremains analysis in the field of palaeopathology. The pathological conditions that may affect the human skeleton are diverse, so here we will focus on conditions that could maximally benefit from the integration of dental calculus data: oral and respiratory health.

#### 3.3.1 Osteoarchaeological evidence on past health and disease

Palaeopathology examines the expression of disease in past individuals and groups by analysing human skeletal remains, mummified tissues as well as parasite eggs. The diseases that may be identified through palaeopathological analysis have various etiologies and manifestations and cover, among others, infectious, metabolic, congenital, circulatory, and endocrine disorders, arthropathies, neoplasms and trauma (Buikstra, 2019) as well as parasitic infection (e.g. Ledger et al., 2019). Like osteoarchaeology more broadly, palaeopathology adopts a biocultural approach whereby skeletal (and other) evidence of disease is viewed within its broader natural and socio-cultural context. This is particularly relevant in palaeopathological analysis since health and disease are largely controlled by the environment that an individual experiences (dietary quality, crowding conditions/exposure to pathogens, daily activities, climate, migration etc.) (Roberts, 2016).

The methods employed in palaeopathology include the macroscopic observation of skeletal lesions, high-resolution microscopy (e.g. scanning electron microscopy), as well as ancient DNA to identify pathogens (De Boer et al., 2013; Grauer, 2007; Miller et al., 1996; Roberts and Ingham, 2008). Despite the wealth of provided information and the availability of a broad range of methods, palaeopathological analysis has a number of limitations that should be taken into consideration in interpreting relevant data. Firstly, the concept of health itself is problematic as it is difficult to effectively measure physical, mental, and social well-being and assess how “healthy” an individual is (Reitsema and McIlvaine, 2014; Temple and Goodman, 2014). Very importantly, many pathological conditions and physiological stressors do not leave traces on the human skeleton, and many others generate very similar skeletal lesions, which hinders differential diagnosis (Buikstra, 2019). Another serious limitation of palaeopathological analysis has been deemed the “Osteological Paradox” (DeWitte and Stojanowski, 2015; Wood et al., 1992). This ‘paradox’ refers to three issues that limit palaeopathological interpretation: (1) demographic nonstationarity, (2) hidden heterogeneity in frailty, and (3) selective mortality. *Demographic nonstationarity* refers to the fact that pre-industrialized groups were “non-stationary,” that is, they were affected by migration, their fertility and mortality rates changed, and there was no equilibrium in age distribution. In such groups fertility exerted a much stronger effect on the age-at-death distribution than mortality. *Hidden heterogeneity* expresses the fact that any skeletal assemblage includes individuals who had different susceptibility to disease. *Selective mortality* is also related to heterogeneous frailty and expresses the fact that the skeletal assemblages under study represent only the individuals who died at any specific age interval, not the entire population. Thus, skeletal assemblages represent the individuals with the highest frailty and overestimate the prevalence of pathological conditions in the entire population.

#### 3.3.2 Linking plant material in dental calculus to medical practices for relieving the symptoms of diseases

Evidence of the deliberate consumption of medicinal plants has been retrieved in dental calculus from Neanderthals (Hardy et al., 2012) through Roman and Medieval times (e.g. Fiorin et al., 2019; Gismondi et al., 2020). The detection of medicinal plants in the archaeological record is challenging. Many species that are medicinal have beneficial properties only if eaten in large quantities and only if the specific medicinal part of the plants is consumed, while the vegetative part often does not survive well. Another problematic aspect of medicinal plants is that they are not routinely consumed by large numbers of people or in high frequencies (unless used in the treatment of chronic diseases). The challenges encountered in archaeobotany to identify such evidence are numerous (see review in Chaves and Reinhard, 2006). An example of these challenges is the consumption of sorrel, a member of the dock Family. This plant was thought to have medicinal properties against plague (Hatfield, 2007, 321). Seeds of sorrel are very common in the archaeological record, but their presence in the archaeobotanical record can be for the most part explained by the fact that sorrel grows easily in disturbed soil and it is a very common crop weed, while it is equally likely that dock leaves and seeds were consumed on a regular basis. Thus, finding such species in an archaeological sample in the Later Medieval period, when episodes of plague spread across Europe, is unlikely to be linked to their use as a remedy but to their wide distribution in the environment.

Similar consideration must be given when evidence of the use of medicinal plants is found in calculus. Dental calculus cannot reveal the quantity of the plant consumed and how often it was taken, for reasons examined before in this paper (see also Radini et al., 2017), and it is not possible to know when the plant was consumed during life. Another aspect to consider is that the same plant may be consumed for the relief of symptoms of multiple pathologies; many of which may not be visible skeletally. To understand these issues better, let us examine two recent cases where plant materials were linked to medical practice. Fiorin et al. (2019) retrieved evidence of species of the fern *Asplenium trichomanes* in the form of the sporangium annulus - a plant and part of the plant that was used in the Middle Ages in Spain to help relieve kidney stones and even alopecia. The authors conducted an in depth historical research and were able to prove there is no attested record for the culinary use of this plant, strengthening the hypothesis that the plant was consumed as medicine. More recently, Gismondi et al. (2020) retrieved by gas-chromatography mass-spectrometry various compounds of medicinal plants and herbs in dental calculus samples from the skeletal remains of a female with the first documented case of coeliac disease. In particular, the authors retrieved *Curcuma* sp. and *Panax* sp., suggesting they were used as relief of the pathology. However, while these plant species were used as medicines, they were also used in Roman and Medieval cuisine. Together with these considerations, we should add that a variety of natural plants may enter the mouth in flecks of soil. Therefore, it should always be cautiously assumed that plants with a medical use identified in calculus were indeed used as ‘medication’ even if the disorder they were taken for is visible on the skeleton. Before closing, we should mention that recent work adopting ultra-high-performance liquid chromatography-tandem mass spectrometry using pneumatically assisted electrospray ionization (UHPLC-ESI-MS/MS) on modern samples begins to reveal the potential of using dental calculus to detect the consumption of drugs and medication in the form of chemical compounds (Sørensen et al., 2021). This is a promising approach though still at an early stage.

The analysis of dental calculus material to study medicinal practices in the past is a venue that needs to be further exploited, especially since at the moment the only osteoarchaeological evidence of past medical intervention comes from ‘invasive’ practices such as trephinations (Nikita et al., 2013), amputations (Micarelli et al., 2018) and other surgical procedures (Redfern, 2010), or from highly indirect evidence (e.g. the long-term survival of individuals with serious disease, which implies some sort of palliative care; see ‘bioarchaeology of care’ approach) (Beckett and Conlogue, 2019; Tilley and Schrenk, 2017).

#### 3.3.3 Oral health in osteoarchaeology and the contribution of dental calculus

The study of pathological conditions of the oral cavity can provide important information regarding past diet, food processing, oral hygiene, and the use of teeth as tools. Even though dental diseases are among the most common conditions recorded in osteoarchaeological studies, there is no consensus as to which conditions may actually be classified as pathological conditions of the oral cavity. In this direction, recent work by Pilloud and Fancher (2019) initiated a discussion towards integrating bioarchaeology with clinical research and standardizing terminology. According to the authors, the conditions classified as dental diseases include dental caries, periodontal disease, periapical lesions, trauma and cancers of the oral cavity. *Dental caries* is expressed as a gradual destruction of the dental hard tissues by organic acids produced during the bacterial fermentation of carbohydrates (Larsen, 2015; Lukacs, 2012; Temple and Larsen, 2007). This is why, as mentioned above in the section on diet, there is a strong link between the expression of dental caries and diet (i.e., the amount of consumed carbohydrates). Beyond diet, caries expression is strongly affected by tooth morphology, age, sex, salivary flow rate, oral hygiene, and other factors (Fakhruddin et al., 2019; Featherstone, 2008). *Periodontal disease* is actually a group of inflammatory diseases that affect the periodontium and eventually result in a reduction of the alveolar crest height (Williams, 1990). It is caused by microbes that form a biofilm on tooth surfaces and generate an inflammatory response. Additional factors that affect the expression of periodontal disease include diet, oral hygiene, hormones, stress, obesity, trauma, and many others (Genco and Borgnakke, 2013; Strohm and Alt, 1998). Clinical studies have found an association between periodontal disease and diabetes, cardiovascular and pulmonary diseases (Beck et al., 1996; Nguyen et al., 2020; Rastogi et al., 2019). *Periapical lesions* manifest on the skeletal tissue around the apex of the tooth root and may be attributed to a granuloma, cyst, or abscess (Dias and Tayles, 1997). *Trauma* to the oral cavity may be acute (e.g. mandibular or maxillary fractures) or chronic (e.g. issues with the temporomandibular joint) (Pilloud and Fancher, 2019). *Cancers* of the oral cavity rarely affect the skeletal tissue; the most common cancer here is squamous cell carcinoma, but it may not leave osseous traces (Chi et al., 2015; Liao et al., 2008). Other conditions that may be included among the dental diseases are dental calculus, hypercementosis and antemortem tooth loss (Pilloud and Fancher, 2019). *Dental calculus* and its etiology have been described above in the section on osteoarchaeological dietary reconstructions. Here we should stress that according to Pilloud and Fancher (2019) calculus is more a product of disease processes rather than the cause of disease, thus, it may be best included as part of pathological conditions of the oral cavity or as a diagnostic trait of periodontal disease. *Hypercementosis* is an excessive buildup of cementum (García-González et al., 2019). Its etiology is unknown but it has been linked to Paget's disease, genetic mutations, thyroid goiter, and other factors (Mohan, 2014; Rao and Karasick, 1982; Thumbigere-Math et al., 2018). Due to its unknown etiology, Pilloud and Fancher (2019) suggest not to include it among dental diseases, but to record it as a dental anomaly. Finally, *antemortem tooth loss* should also be used cautiously as it is multifactorial in etiology and some causative factors are not related to disease (e.g., dental ablation) (Lukacs, 2012).

The key evidence for oral health and pathologies retrieved from human dental calculus so far is of biomolecular nature, and includes the study of the oral microbiomes and opportunistic oral pathogens, with technological advances in this field continuously improving (e.g. Adler et al., 2013; Velsko et al., 2019; Warinner et al., 2014). Wright et al. (2021) have stressed that research is revealing the link between the genetics of the oral microbiota and that of the host. Links have also been made between diet, food processing technologies and the oral microbiomes (Adler et al., 2013). However, little has been said about the influence of particles that enter the human mouth by accidental ingestion or through the use of the mouth as a third hand, and their effect on dental wear and oral hygiene, which can also influence the microbiome. Here we provide examples of particulate matter that could affect the oral cavity and may play an important and underestimated role in our understanding of oral health.

**Fig. 2:**
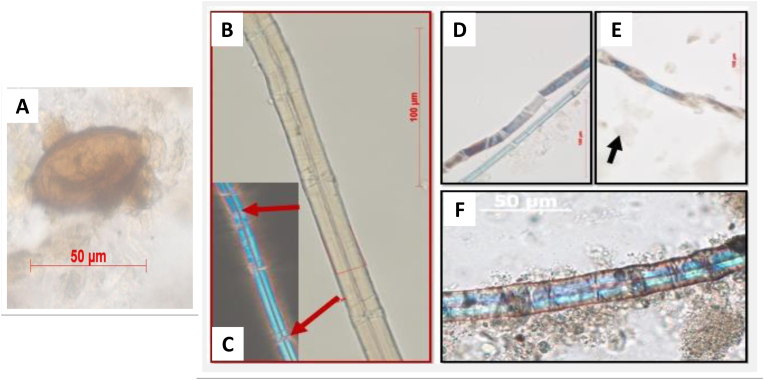
**A:***Trichuris* sp. egg. Note the egg is still embedded in the calculus matrix (the cloudy nature of the image is due to the dissolving calculus matrix in HCl). The egg is also surrounded by amorphous organic matter, very likely humic substance from soil. **B–C**: Flax from the reference collection. **D-F**: Examples of bast fibers from calculus matrix. C is an example of fiber that showed dislocation bands. In E the fiber is ‘twisted’ and, finally, F shows a fiber identified as flax based on the clear dislocation band, the narrow lumen and the optical properties - note the similarities with the reference material (the arrow in E points to the fleck of calculus separating from the fiber). All pictures by Radini (2016).

A variety of dental calculus finds, not directly associated with food consumption, points to the possibility of investigating oral and food hygiene. Such finds include for example fungal spores (Fig. 1 B), hyphae and yeast spores (e.g. Fiorin et al., 2019; Hardy et al., 2017; King et al., 2017a; Power et al., 2015; Radini et al., 2017). These are known to be difficult to be taxonomically identified (Li et al., 2007, 98), but nonetheless they are likely creating an unfavorable oral environment. Similarly, remains of soil and grit systematically found in dental calculus deposits could enhance dental wear and irritate the soft tissues, rendering teeth more prone to oral diseases (e.g. Buckley et al., 2014; Cristiani et al., 2016; Hardy et al., 2018; Radini et al., 2017). Microcharcoal and soot are also nearly ubiquitous in dental calculus samples dating from nearly half a million years ago (Hardy et al., 2016), supporting that the oral environment hosts in the long-term several microparticles that could be deleterious to the health and contribute to dental wear, like mineral grit (e.g. Radini, 2016). Finally, even if they are rare findings, previous studies have retrieved evidence of intestinal parasites in dental calculus (Fig. 2 A), coming either from contaminated soil or food (Radini, 2016) as well insect parts (Cristiani et al., 2016; Hardy et al., 2016; MacKenzie et al., in press). All the above lines of evidence, especially if combined, support that dental calculus can be also seen as a potential reservoir of information on microparticles directly or indirectly linked to oral and food hygiene. Given the complex and multifactorial etiology of oral diseases, it is paramount to consider any evidence on oral hygiene provided by calculus; thus, all lines of evidence found in calculus should be reported, even if it is not possible to identify them.

#### 3.3.4 Respiratory health and built environments

Probably one of the most exciting new ventures of analysis in dental calculus is that associated with exposure to respiratory irritants of natural and anthropogenic origin, which may provide important background environmental information to respiratory disorders, often of multi-causal origin.

The two main respiratory conditions that have been examined in osteoarchaeological assemblages are sinusitis and lower respiratory tract disease. Sinusitis is an infection of the mucous membrane of the paranasal sinuses, which may spread to the maxillary sinuses. It may be caused by bacteria, viruses or fungi and often manifests skeletally as new bone formation or bone resorption (Rosenfeld, 2016; Sethi, 2015; Slavin et al., 2005). Several osteoarchaeological studies have identified sinusitis in various time periods and geographical regions (Krenz-Niedbała and Łukasik, 2016; Lewis et al., 1995; Merrett and Pfeiffer, 2000; Sundman and Kjellström, 2013). Multiple factors have been linked to the expression of sinusitis, such as upper respiratory tract infections, dental infections, trauma, asthma, immunodeﬁciency diseases, or environmental conditions such as pollution, overcrowding, low humidity, poor air quality, smoke, or cold and damp climates (Berlinger, 1985; Khanna and Gharpure, 2012; Melén et al., 1986; Newman et al., 1994; Troeltzsch et al., 2015). Lower respiratory tract disease has primarily been identified osteoarchaeologically as an inflammatory periosteal reaction on the visceral surface of the ribs (Davies-Barrett et al., 2019). It has been proposed that inflammation within the pleural cavity is what stimulates periosteal reaction on the visceral rib surfaces (Kelley and Micozzi, 1984; Roberts et al., 1994, 1998). In clinical contexts, tuberculosis, pneumonia, and actinomycosis are the commonest respiratory infections associated to pleural inflammation (Kass et al., 2007). In osteoarcheological contexts, lower respiratory tract diseases and poor air quality (e.g., badly ventilated spaces and exposure to particulate pollution) may be concluded through the presence of rib periostitis (Capasso, 2000; Lambert, 2002; Roberts, 2007). However, determining the etiology of rib lesions in archaeological populations is complicated by the range of causes of pleural disease that are unrelated to lower respiratory tract infection, such as malignancy, heart and abdominal diseases (Rahman et al., 2004, 31).

Before entering into the discussion of the typologies of debris of interest in this section, some necessary background information and clarification of terminology needs to be provided. Various microdebris in dental calculus may be interpreted as ‘dust’ present in the environment accidently ingested or inhaled. In this paper we will use the term ‘dust’ as the assemblage of particles of various nature, of organic and inorganic origin, of different size and shape, that are normally present in the atmosphere around us. Dust has a biological (e.g., pollen, spores, fibers, insect parts, dust mites and bacteria) and a lithological component (i.e., mineral fragments of natural and anthropogenic origin), and can also contain smoke, microcharcoal and soot. Dust is normally divided into airborne and settled dust, where airborne dust is normally below 100 μm in size. Here the main focus is on just one category of dust known as ‘particulate matter’ (PM). PM consists of tiny fragments of airborne debris that is, at least for part of its life, ranging in size from below 1 μm up to at least 100 μm. As noted by Yang and Omaye (2009), particles larger than 10 μm in diameter rarely reach and settle in the lungs; particles smaller than 10 μm can reach the large upper branches; particles smaller than 5 μm can reach the bronchial tubes, and particles smaller than 2.5 μm can enter the alveolar parts of the lung. For this reason, particles below 10 μm are often called ‘respirable PM’. Fine particles (between 2.5 μm and 0.1 μm in diameter) and ultrafine particles (diameter smaller than 0.1 μm) can enter the bloodstream if soluble in water (Yang and Omaye, 2009). It should be stressed that studies have proven that the diameter of the particles, not their overall size, is the most important factor determining whether they will reach the lungs (Pope and Dockery, 2006). This explains why fibers as long as 100 μm, with diameter below 5 μm have been found in the lower respiratory system. Such aspects result in occupational dust from certain industries, for example textile work, to be particularly irritating to the respiratory system.

When we consider the variety of remains of non-dietary origin in dental calculus, such as microcharcoal, pollen, fungal spores, bast fibers (Fig. 2B–F) and even particles of pigments (e.g. Blondiaux and Charlier, 2008; Power et al., 2018; Radini et al., 2019), they mostly fall into the category of ‘respirable fraction of particulate matter’, therefore they are a potential source of information regarding the environment ancient people directly experienced during life. Indeed, though rare, fibers used in textile and other crafts have been retrieved in dental calculus, including bast fibers such as hemp, flax and nettle, cotton (e.g. Cristiani et al., 2016; Radini et al., 2016; Warinner et al., 2014), cotton (Blatt et al., 2011; MacKenzie, 2021), wool (Radini, 2016) and potentially even leather corium (Warinner et al., 2014). Furthermore, the presence of potential respiratory irritants in calculus, such as fungal spores, microcharcoal and soot (e.g., Buckley et al., 2014; Hardy et al., 2016) and even dust mites and wood dust (e.g. MacKenzie et al., in press), is of great interest for understanding exposure to pollution and dusty environments. The potential of detecting respiratory irritants is particularly valuable in disorders such as maxillary sinusitis, which has been linked to exposure to smoke and pollution in many populations (Roberts, 2020). Nonetheless, the association between potential respiratory irritants, such as microcharcoal, and sinusitis, is not straightforward. The multiple pathways of inclusion of microparticles in dental calculus complicate the interpretation of such remains, as they may not be inhaled but accidently ingested, for example as dirt on food. In the case of exposure to smoke, the identification of microcharcoal and soot in dental calculus is not always secure. Particles smaller than 3 μm, often interpreted as soot when appearing as black dots, cannot be securely identified as microcharcoal solely using light microscopy since several inorganic particles resemble microcharcoal under light microscopy (brown to black color, opaque, sharp angular edges) (Blackford, 2000; MacKenzie et al., in press). Equally, the origin of such particles may not necessarily be ‘inhalation’ (Radini et al., 2017) as smoke had several uses in the past including food preservation (Pennacchio et al., 2010), so pathways to its inclusion are many (see MacKenzie et al., in press for a more detailed discussion). Furthermore, it is nearly impossible to assess the frequency and intensity of exposure to respiratory irritants based only on the particles found in dental calculus, and prolonged exposure to high concentration of them would be necessary to result in a pathology. Buckley et al. (2021) have tentatively used the size of microcharcoal to assess the nature of the exposure to smoke, however, as shown by Bartholdy and Henry (2021), there are complex interactions between the physical structure of dental calculus and its ability to ‘capture’ microparticles, resulting in a clear selection process in relation to their size. Care must be taken in the interpretation of such results.

Given the inherent limitations in osteoarchaeological and dental calculus research, the joint study of macroscopic skeletal lesions potentially associated with upper or lower respiratory tract disease and dental calculus dust microparticles, could potentially offer a more comprehensive understanding of respiratory irritants and their health implications. Steps in this direction have been initiated, such as the identification of wool fibers in the dental calculus of individuals with evidence of tuberculosis (Rossi et al., 2020). While linking such evidence to ‘high exposure’ to respiratory irritants should be cautious, the combination of dental calculus micremains and osteoarchaeological parameters may strengthen the interpretation of the remains in calculus, while providing complementary evidence to pathologies. Further work on this topic is urgently needed, however a step forward has been recently made by Buckley et al. (2021), who were able to detect, for the first time the inhalation of fire-smoke from lignite using Thermal Desorption/Pyrolysis–Gas Chromatography-Mass Spectrometry in dental calculus from the Eastern Mediterranean dating 2nd millennium BCE.

### 3.4 Occupation

Partly linked to the theme of respiratory health, is the final topic explored in this paper, namely the examination of past occupations by integrating osteoarchaeological and dental calculus approaches. Occupation is a characteristic that largely defines social status, health and other aspects of an individual's biosocial identity, whilst at the same time being among the most elusive aspects osteoarchaeologically (Jurmain et al., 2012). This section will focus on crafting activities, which broadly represent the occupation of a large proportion of past populations from the Bronze Age onwards.

#### 3.4.1 Evidence of crafting activities on human skeletal remains

Past activities leave marks on skeletal and dental remains and the key methods used to explore these are entheseal changes (Karakostis et al., 2019), osteoarthritis (Eng, 2016), and cross-sectional geometric properties of the long-bone diaphyses (Nikita et al., 2011). A serious limitation of all available methods is that they are non-specific in the sense that they may suggest that one individual was more robust than another or suffered from more intense mechanical stress; however, they cannot pinpoint the exact activities in which this individual engaged. This issue is linked to the fact that different activities may generate similar functional stresses and these stresses may affect differently specific individuals (Jurmain et al., 2012). In addition, entheseal changes and osteoarthritis are multifactorial in etiology, with age and body size generally playing a more important role in their expression compared to activity (Michopoulou et al., 2015; Myszka et al., 2020, but see Karakostis et al., 2017, 2019).

Despite these limitations, a number of recent studies have attempted to explore the skeletal evidence that specific crafting activities may have left on past individuals, an approach largely promoted by the interest of modern osteoarchaeology in establishing past osteobiographies. A typical example is Becker's (2016) study of a 30–39 years old female (CJ-35250) from the Ch'iji Jawira site in Tiwanaku, Bolivia, to assess whether this individual was involved in pottery manufacture or weaving. The author found that entheseal changes and osteoarthritis on the carpals, metacarpals, and upper limb long bones could indicate involvement in repetitive tasks that require upper body strength and manual dexterity; therefore, they are compatible with both suggested activities. Similarly, the well expressed entheseal changes and osteoarthritis in the mid-body, tarsals, and metatarsals were interpreted as compatible with modern Andean pottery making and weaving practices, which involve the upper and lower limbs, as well as prolonged sitting in awkward positions. In the same direction Yi et al. (2019) focused on the osteobiography of a seventh century potter at the Oupan kiln, China, combining osteological and isotope data. The authors identified vertebral osteophytosis, knee osteoarthritis, and abnormal curvature in the lower thoracic and upper lumbar part of the spine. These skeletal changes could be associated with occupational activities such as pottery making. In addition, the authors used carbon, nitrogen and oxygen isotope analyses and concluded that the individual under study had drastically changed his diet and/or migrated several times throughout his lifetime, which they also linked to the individual's low social status. Similarly, Baker et al. (2012) explored the possibility of a 30–39 years old female buried at Polis Chrysochous, Cyprus, being a seamstress as a bone needle was found with her inside the tomb. The authors highlighted a number of skeletal and dental alterations that are compatible with this female being a seamstress, such as malocclusion and wear in the anterior teeth, well-developed muscle attachments and facets on the hand bones, and alterations to the legs that could be attributed to habitual kneeling or squatting. The results of the above sample studies highlight anew some of the limitations regarding deciphering past occupations from the skeleton: a) skeletal changes are nonspecific: very different activities may produce very similar skeletal responses, b) had we not known in advance the potential range of occupations for these individuals (from contextual data), it would have been impossible to postulate them based on the observed skeletal changes.

#### 3.4.2 Record of crafting activities in dental calculus

A number of finds in dental calculus can be the result of crafting activities, for example microremains of organic origin such as textile fibers (Blatt et al., 2011; Radini et al., 2017) and phytoliths from plants potentially used for basketry, roofing and flooring material, such as *Phragmites australis* (Norström et al., 2019), as well as inorganic particles, such as minerals from pottery clay (Blondiaux and Charlier, 2008) and even pigments (Radini et al., 2019). These lines of evidence are very promising, but currently little explored as most studies focus on dietary remains and often do not report the presence of any other finds besides starch and phytoliths. Despite this limitation, the remains retrieved so far from calculus that can be the result of occupational activities may be divided into two main categories described below.

The first includes microremains where the pathway of inclusion is unequivocally the result of an occupational activity. In a typical case study, lapis lazuli ‘dust’ was retrieved in calculus from a Medieval female individual from Dalheim and linked to manuscript illumination (Radini et al., 2019). The rarity of the material, and the particle size and distribution allowed the authors to exclude other pathways of inclusion besides that of manuscript illumination. This was not possible for other ‘coloured’ particles that could potentially be present in soil, such as hematite. The second category includes microremains such as textile fibers, which may also be present in the environment and, therefore, the link to crafting activities can only be established in combination with other parameters from the skeleton, such as dental wear, in presence of high concentration of debris and were a sufficiently large number of individuals are available to allow statistical analysis. This is the case for cotton, flax and nettle fibers found in a number of case studies across different periods of time (e.g. Cristiani et al., 2016; MacKenzie, 2021; Radini, 2016). In previous studies, such remains were identified in low quantities and could be the result of accidental ingestion/inhalation of dust in the environment. Moreover, it must be stressed that some finds, such as those of common reed (*Phragmites australis*), could be the result of multiple activities, so it may never be possible to narrow the pathways of their inclusion down to a single one. Finally, in studies where concentration of remains and their size is crucial in their link to craft and exposure to pollutants, understanding the topography and structure of dental calculus is crucial. In any case, dental wear and other osteoarchaeological parameters could strengthen the link between debris and craft.

It is clear from the overview above that dental calculus captures debris that could be generated by crafts practiced at monumental scale in the past, such as textile production, stone work and pottery making. Whenever it is possible to narrow down their pathways to inclusion, such remains may be used to track ancient crafters with a resolution not possible before. Experimental Archaeology can also allow to better understand flux of particles of non-dietary origin in the human mouths and their survival and alteration in human saliva (Radini et al., 2019). It is expected that further work on accidental ingestion and oral breathing of both particulate matter and chemical compounds during crafting activities will be a promising venue in this field. Finally, experimental work is in fact showing that dental calculus surface and porosity can affect the size of particulate matter that become entrapped in its matrix (Bartholdy and Henry, A.G., 2021).

### 3.5 Life course approaches

Life course approaches view individuals as the sum of previous social and biological life experiences, and they examine them in connection to their historical and sociopolitical contexts. Hence, such approaches emphasize the interconnectedness of biological and social processes, and of different life stages (Mitchell et al., 2015; Quante et al., 2012). In bioarchaeology, life course approaches have been adopted to explore issues of development, physiological stress, mobility, diet, trauma and others (e.g. Agarwal, 2016; Cerezo-Román and Tsukamoto, 2021; Glencross, 2011; Gowland, 2015; Lockau et al., 2019). Here we will examine the topic of diet as this is a key area where the integration of dental calculus data could be highly beneficial.

Food is essential for our biological survival but at the same time food access may change through one's life course and depends on historical and socio-political factors. Therefore, dietary studies are a prime area for the application of the life course approach. In this direction, studies have combined isotope data from different skeletal and dental elements of the same individual to assess dietary changes over the life course (e.g. Beaumont and Montgomery, 2016; Reitsema and Vercellotti, 2012). These studies are based on the fact that different dental and skeletal tissues form at different stages in an individual's life and have different remodeling rates (e.g. tooth enamel does not undergo remodeling, while bone is remodeled throughout life in response to physiological needs and biomechanical stress). Similarly, incremental dental sampling methods (Beaumont et al., 2013; Craig-Atkins et al., 2018; Henderson et al., 2014) can identify dietary change throughout an individual's early development (when the dental tissues formed) and are being increasingly adopted in studies of breastfeeding and weaning (e.g. Czermak et al., 2018; King et al., 2017b).

Dental calculus data could make an important contribution to life course approaches in bioarchaeology by further refining the available information by noting the presence of particular types of foods in diet, inclusion of particles from the environment, and occupation, as specified in the previous sections. Nonetheless, this potential contribution is hindered by the fact that it is currently not possible to assess what portion of an individual's lifetime is represented in the calculus matrix (e.g. Power et al., 2015). Carefully designed sampling strategies, which take into consideration the age of formation of different deciduous and permanent teeth, could narrow down the time span during which calculus formed and the dietary, occupational, environmental and other information retrieved from the calculus deposit could be linked to a specific period of an individual's life. As an example, one of the authors (A.R.) selected young adults and sampled calculus from the third molars, which are the last teeth to erupt and, even though they exhibit the greatest variation in their eruption timing, they usually achieve full eruption by 23 years (Shaweesh, 2016; Tuteja et al., 2012). In this way, the author ensured maximum comparability of data in the medieval population of Leicester (Radini, 2016). Such an approach can, therefore, be used to narrow down the period in an individual's lifetime represented in the calculus selected for analysis.

## 4 Conclusions

The study of ancient human dental calculus has increased in popularity in the past decades. There has been an intense focus on diet and on specific typologies of microremains, such as starches and phytoliths, entrapped in its matrix. However, increasing evidence is showing that a variety of other remains are captured in this deposit on teeth. Our paper suggests diverse research themes which would benefit the most from an integration of dental calculus analysis with other osteoarchaeological parameters, namely the reconstruction of diet, health, occupation, natural and built environment, exposure to pollutants, and life course approaches. We encourage a dialogue between those studying dental calculus and those specialised in the study of ancient human remains in order to rethink research questions and maximize the interpretational potential of both lines of evidence. We acknowledge that many of the research topics introduced can only be explored where preservation of remains and number of skeletons are large, as is the case for more recent periods of time, where historical records can also strengthen the interpretation. Nonetheless, this fast moving venue of analysis is in urgent need of more effective integration with other lines of evidence in order to enhance its potential, and human skeletal remains are an excellent starting point given their strong contextual link with dental calculus.

## Author contributions

AR with EF conceived the work. AR and EF wrote the paper.

## Data availability

Any unpublished data mentioned in the text is available upon request.

## Declaration of competing interest

The authors declare that they have no known competing financial interests or personal relationships that could have appeared to influence the work reported in this paper.

## Footnotes

1 The term which denotes the study of human remains in US contexts is ‘bioarchaeology’, while ‘osteoarchaeology’ is the preferred term in European contexts, where bioarchaeology signifies the study of all organic remains. Note that the term ‘osteoarchaeology’ also encompasses the study of animal skeletal remains; however, in the current paper it is used solely to indicate the study of human material.
